# Variation in Intramuscular Fat Deposition of Goats and Sheep and Its Correlation with Gut Microbiota

**DOI:** 10.3390/foods14111885

**Published:** 2025-05-26

**Authors:** Lei Yang, Shaobin Li, Jiagong Hou, Zhisheng Tang, Bingang Shi, Yuzhu Luo, Jiqing Wang, Fangfang Zhao

**Affiliations:** 1 Gansu Key Laboratory of Herbivorous Animal Biotechnology, Faculty of Animal Science and Technology, Gansu Agricultural University, Lanzhou 730070, China; 13195822513@163.com (L.Y.); lisb@gsau.edu.cn (S.L.); 17793252927@163.com (Z.T.); shibg@gsau.edu.cn (B.S.); wangjq@gsau.edu.cn (J.W.); 2 Jinan Zhangqiu District Agriculture and Rural Affairs Bureau, Jinan 250200, China; songsong.888@163.com

**Keywords:** IMF deposition, Longdong cashmere goat, Tan sheep, lipid levels, lipid-metabolizing enzymes, SCFAs, gut microbiota, correlation

## Abstract

The meat quality of sheep and goats differs even within the same age, gender, and farming systems. Intramuscular fat (IMF) content is an important factor affecting the quality of livestock meat because it affects muscle color, tenderness, juiciness, water-holding capacity, and flavor. This study evaluates the differences in IMF deposition characteristics between Longdong cashmere goats and Tan sheep, and also explores the correlations between these variations and the gut microbiota. The results revealed that the IMF contents in shoulder and rump meat, as well as the blood lipid levels, of Longdong cashmere goats were higher than those of Tan sheep (*p* < 0.05). The content of fatty acid synthase (FAS) in the duodenum of the goats was lower, but the content of hormone-sensitive lipase (HSL) in both the pancreas and duodenum was greater (*p* < 0.05). The Chao1 and β diversity showed differences between the two breeds, observed not only in the abomasum but also in the colon. The specific microbiota identified from the goats were involved in the lipid metabolism pathway. The concentrations of acetic acid and propionic acid in the colonic and abomasal chyme were decreased in the goats when compared to the sheep (*p* < 0.05). The contents of FAS in the colonic chyme of the goats were significantly lower, while HSL in the abomasal chyme was significantly higher than that of the sheep. The correlation analysis of IMF deposition with gut microbiota showed that *Acetobacter* and *UBA1711* in the abomasum, as well as *Faecousia*, *WQUU01*, *UBA5905,* and *GCA-900066495* in the colon, were positively correlated with the IMF content in shoulder meat and the level of LDL (except for *UBA1711*), but negatively associated with the content of propionic acid (|r| > 0.45, *p* < 0.05). This preliminary study has demonstrated that some specific bacteria in the abomasum and colon were associated with IMF deposition, while also providing an indicative reference range for further investigation into the effects of microbes on IMF deposition.

## 1. Introduction

The global production of goat and sheep meat has experienced a significant increase of 57.83%, rising from 78.5 million tons in 2000 to 123.9 million tons in 2023 [1]. With the enhancements in material living standards, the demand for high-quality meat is increasing, but meat-eaters are placing greater emphasis on the tenderness, taste, and flavor of meat. Varying levels of intramuscular fat (IMF) contribute to different degrees of ‘marbling’ in the muscle, which affects tenderness, flavor, juiciness, and other meat quality indicators [2,3]. For example, one study revealed that IMF can weaken the association between collagen fibers, making muscle fibers more likely to break during chewing and thereby enhancing muscle tenderness [4,5]. Gaining a deeper understanding of the physiological patterns of IMF deposition in sheep and goats contributes to the development of more effective strategies aimed at enhancing meat quality and satisfying the increasing consumer demand for both health and flavor attributes.

Studies have documented the regulatory mechanisms of IMF deposition at the biochemical [6], molecular [7], and cellular levels [8]. These studies have revealed that enzymes and transcription factors play a crucial role in regulating adipocyte differentiation and adipogenesis. This understanding is significant for enhancing the meat quality. In recent years, advancements in metagenomic sequencing technology have led to an increasing number of studies revealing that the composition and structure of microbiota in the digestive systems of livestock influence IMF deposition. For example, Zeng et al. [9] found a correlation between the abundance of *Bacteroides*, *RuminococcaceaeP7*, *Eubacterium ruminantium*, and *Prevotella* in the rumen and the fatty acid content of the *Longissimus dorsi* muscle in Hechuan white goats. Adding *Clostridium butyricum* to the diet can enhance the IMF content in the leg muscles of broiler chickens [10], and the presence of *Klebsiella* and *Escherichia-Shigella* in the intestines of yellow chickens is associated with increased levels of total cholesterol (TC) and triglycerides (TGs) in the blood, which contributes to fat accumulation [11]. Additionally, the impact of gut microbiota on host fat accumulation may occur through microbial metabolites, such as short-chain fatty acids (SCFAs), which can influence host lipid metabolism by modulating nutrient detection, nerve signal transmission, and hormone release within the digestive system [12].

To date, there is a paucity of research investigating the potential relationship between variations in fat deposition among sheep and goats and their gut microbiota. Investigating the specific microbiota associated with superior meat quality characteristics is of considerable importance for enhancing the production of high-quality lamb. For example, Wang et al. revealed that the rescheduling of the gut bacterial community contributed to imoroved meat quality in Tan lambs grazing on artificial pastures [13]. This approach offers a potential option with which to produce healthier lamb meat products, but few microbes associated with fat deposition have been identified in sheep and goats. Longdong cashmere goats and Tan sheep are indigenous breeds found in China, each exhibiting unique flavor profiles [14,15]. This study co-grazed these two species to investigate their differences in IMF deposition characteristics and gut microbial composition. Through a correlation analysis of variation characteristics and identified specific bacteria, we identified some potential bacteria and SCFAs associated with IMF deposition. This finding provides a reference range for subsequent experiments on manipulating IMF content through microbial colonization or probiotic supplementation.

## 2. Materials and Methods

### 2.1. Animals

This study focused on 1-year-old Longdong cashmere goats (*n* = 10) and 1-year-old Tan sheep (*n* = 10), both female, co-grazing in Huan County, Qingyang City, Gansu Province, and all of them originated from the same herd and grazing group.

### 2.2. Sample Collection

Jugular vein blood was collected from each animal. After centrifugation, serum samples were obtained for the determination of lipid-related indicators.

Prior to slaughter, the live weight was recorded. After slaughter, the hot carcass weight was measured, and a GR carcass fat assessment was undertaken by measuring the tissue thickness (mm) at the interval between the 12th and 13th ribs, 11 cm below the midline of the back. The dressing percentage was calculated as the ratio of hot carcass weight to live weight. Muscle tissues from the shoulder, *Longissimus dorsi*, rump, and rib chops were collected to measure the IMF content. The tissues of the liver, pancreas, and duodenum, as well as gut chyme from the rumen, abomasum, and colon, were collected. All samples were immediately frozen in liquid nitrogen and then stored at −80 °C until use.

### 2.3. Determination of IMF Content, Concentrations of Blood Lipid, Enzyme Levels, and Fatty Acid Content

The IMF content of the muscle tissue samples was determined by using Soxhlet fat extraction (ANKOM XT15 Extractor, ANKOM Technology, Macedon, NY, USA).

The serum samples were used to determine the concentrations of high-density lipoprotein (HDL), low-density lipoprotein (LDL), very-low-density lipoprotein (VLDL), free fatty acids (FFAs), TG, and TC using HDL, LDL, VLDL, FFA, TG, and TC ELISA kits (Shanghai Enzyme-linked Biotechnology Co., Ltd., Shanghai, China).

The contents of fatty acid synthase (FAS) and hormone-sensitive lipase (HSL) in the liver, pancreas, and duodenum, as well as gut chyme, were determined using a FAS ELISA kit (Nanjing Jiancheng Bioengineering Institute, Nanjing, China) and HSL ELISA kit (Shanghai kexing Trading Co., Ltd., Shanghai, China), respectively.

The contents of SCFAs in the gut chyme were analyzed using the method described by Tangerman [16] with an Agilent 6890 N Network Gas Chromatograph (Agilent Technologies, Santa Clara, CA, USA).

### 2.4. SrRNA Analysis

#### 2.4.1. Microbial DNA Extractions and PCR Amplification

Microbial DNA was extracted from the goat and sheep intestinal content samples by using an E.Z.N.A.^®^ Stool DNA Kit (Omega Bio-tek, Norcross, GA, USA) according to the manufacturer’s protocols. The full-length bacterial 16S ribosomal RNA gene was amplified via PCR using the primers 27F (5′-AGRGTTYGATYMTGGCTCAG-3′) and 1492R (5′-RGYTACCTTGTTACGACTT-3′), and a barcode that was an eight-base sequence unique to each sample [17].

#### 2.4.2. Library Construction and Sequencing

SMRTbell libraries were prepared from the amplified DNA via blunt-end ligation following the manufacturer’s instruction (Pacific Biosciences, Menlo Park, CA, USA) and sequenced on a dedicated PacBio Sequel II platform by using Sequencing Kit 2.0 chemistry (Pacific Biosciences of California, Inc., Menlo Park, CA, USA).

#### 2.4.3. Processing of Sequencing Data

PacBio raw reads were processed using SMRT Link Analysis software (version 9.0) to obtain de-multiplexed circular consensus sequence reads. Raw reads were processed through SMRT Portal to filter sequences for length (<800 or >2500 bp) and quality. Sequences were further filtered by removing barcodes, primer sequences, chimeras, and any sequences that contained 10 consecutive identical bases. Operational taxonomic units (OTUs) were clustered with a 98.65% similarity cutoff using UPARSE (version 7.1), and chimeric sequences were identified and removed using UCHIME [18]. The phylogenetic affiliation of each 16S rRNA gene sequence was analyzed by the UCLUST algorithm (v1.2.22q) [19] against the Greengenes2 16S rRNA database with a confidence threshold of 80% [20]. The raw reads were deposited into the NCBI Sequence Read Archive (SRA) database (accession number: PRJNA1214480).

#### 2.4.4. Alpha- and Beta-Diversity Analyses

The Chao1 and Shannon indices were conducted to estimate species richness in a community. A principal coordinate analysis (PCoA) and non-metric multidimensional scaling (NMDS) plotting analysis were performed to estimate beta diversity.

#### 2.4.5. Functional Prediction of the Microbial Genes

The Phylogenetic Investigation of Communities by Reconstruction of Unobserved States (PICRUSt2) [21] program, based on the Kyoto Encyclopedia of Genes and Genomes (KEGG) database, was used to predict the functional alteration of microbiota in different samples. The OTU data obtained were used to generate BIOM files formatted as input for PICRUSt2 with the makebiom script usable in the ‘mothur’ [22]. The OTU abundances were mapped to Greengenes2 OTU IDs as input to assess the functional alteration of microbiota.

### 2.5. Statistical Analysis

The independent samples *t*-test was performed by using SPSS software (version 20.0) to compare differences in carcass quality (dressing percentage and GR), IMF content, lipid levels, lipid metabolism enzymes, and the contents of SCFAs between the Longdong cashmere goats and Tan sheep. Differences were considered significant at *p* < 0.05. The Chao1 and Shannon diversity indices were analyzed based on the abundance of each OTU in each sample by using the “vegan” package in R (version 4.1.0). The difference in the alpha diversity between the four groups was compared by using Kruskal–Wallis test. PCoA was performed based on the Bray–Curtis distance matrix among samples by using the ape package [23]. One-way permutational analysis of variance (PERMANOVA) was performed to assess the statistical significance of differences between groups. The NMDS plotting analysis was performed based on the Unweighted UniFrac distance matrix by using the ‘vegan’ package [24]. The differences in microbial genera were assessed using Kruskal–Wallis test, while the Wilcoxon rank-sum test was used for the statistical analysis of various functional pathways. Spearman correlation coefficients were assessed to determine the relationships between age and chemical factors, microbiota, and contents of IMF and SCFAs. Differences were considered to be significant at *p* < 0.05.

## 3. Results

### 3.1. The Differences in Dressing Percentage, GR Value, and IMF Content of the Goats and Sheep

**Figure 1:**
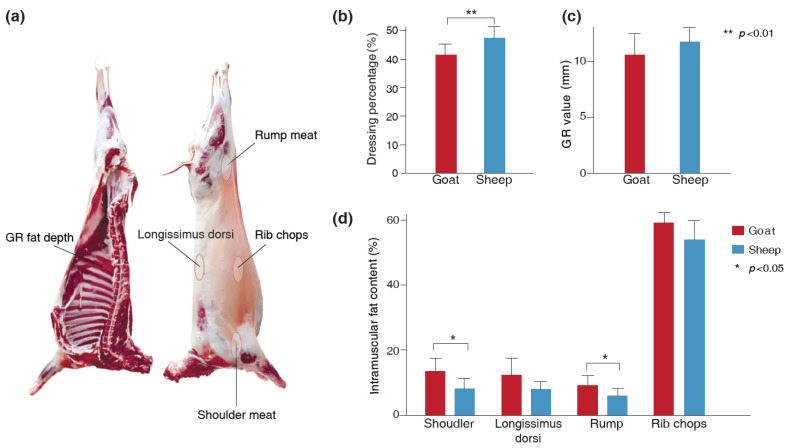
The differences in dressing percentage, GR value, and IMF content of the goats and sheep. (**a**) The location of the meat tested. The GR value is considered as the tissue thickness between the 12th and 13th ribs, 11 cm from the midline of the dorsal spine. (**b**) Difference in the dressing percentage of the goats and sheep. (**c**) Difference in the GR value of the goats and sheep. (**d**) Difference in the meat IMF content of the goats and sheep.

To evaluate the impact of species on carcass traits and meat quality, we determined the dressing percentage, GR value, and IMF content in shoulder meat, *Longissimus dorsi* muscle, rump meat, and rib chops of Longdong cashmere goats and Tan sheep. The results revealed that the dressing percentage of Tan sheep was higher than that of Longdong cashmere goats (*p* = 0.003), while the IMF content in shoulder meat (*p* = 0.02) and rump meat (*p* = 0.04) was lower (Figure 1). No significant differences were observed in the GR value and the IMF content of the *Longissimus dorsi* muscle and rib chops between the two breeds (Figure 1).

### 3.2. Variations in the Physiological Indicators of the Goats and Sheep

**Figure 2:**
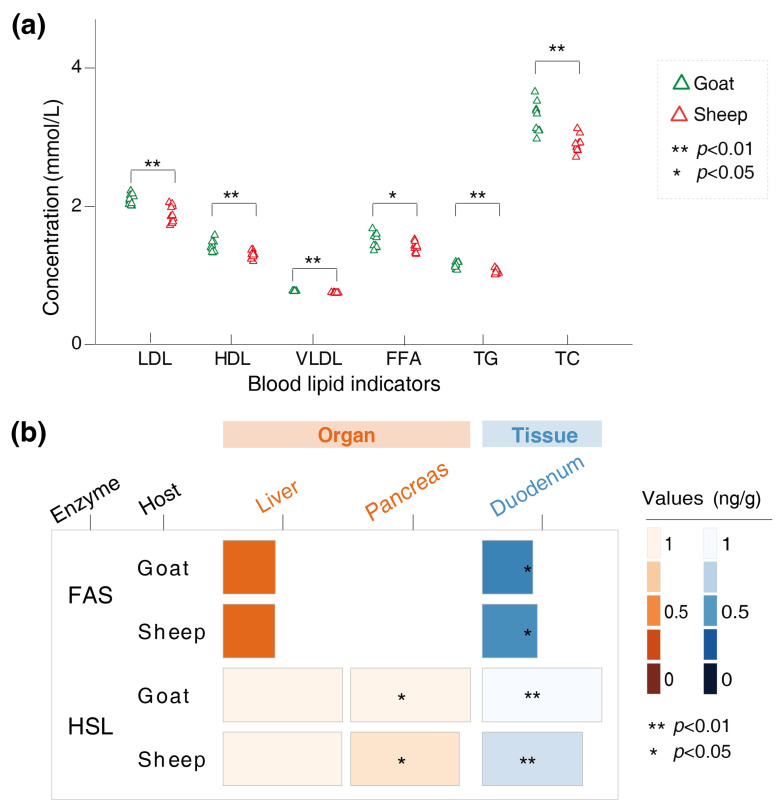
Variations in the physiological indicators of the goats and sheep. (**a**) Differences in levels of LDL, HDL, VLDL, FFAs, TG, and TC of the goats and sheep; (**b**) Differences in the FAS and HSL content in the liver, pancreas, duodenum of the goats and sheep.

We determined the levels of LDL, HDL, VLDL, TG, TC, and FFAs in the serum of Longdong cashmere goats and Tan sheep. The results showed that the levels of LDL (*p* < 0.001), HDL (*p* = 0.008), VLDL (*p* < 0.001), TG (*p* < 0.001), TC (*p* < 0.001), and FFAs (*p* = 0.04) were higher in the goats than in the sheep (Figure 2a). The FAS content in the duodenum of the goats was lower than that of the sheep (*p* = 0.04). In contrast, the HSL content in both the pancreas (*p* = 0.04) and duodenum (*p* < 0.001) of the goats was higher than that of the sheep (Figure 2b).

### 3.3. Differences in the Gut Microbiota of the Goats and Sheep

**Figure 3:**
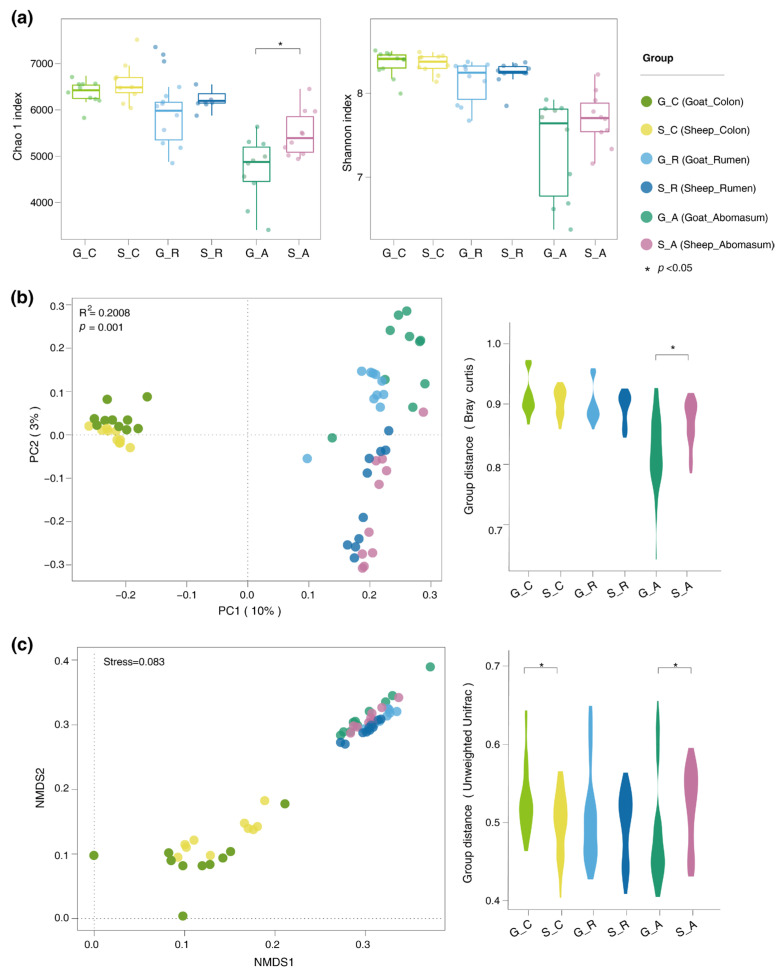
Differences in the gut microbiota of the goats and sheep. (**a**) Comparison of the Chao 1 and Shannon indices of gut microbiota in gastrointestinal contents of goats and sheep; (**b**) PCoA plots of gut microbial community among different gastrointestinal tract of goats and sheep; (**c**) NMDS plots of gut microbial community among different gastrointestinal tract of goats and sheep. GR denotes the ruminal chyme from Longdong cashmere goats, while SR denotes the ruminal chyme from Tan sheep. GA refers to the abomasal chyme of the goats, and SA refers to the abomasal chyme of the sheep. GC represents the colonic chyme of the goats, and SC represents the colonic chyme of the sheep. * represents *p* < 0.05.

The alpha and beta diversity of the gut microbiota in the Longdong cashmere goats and Tan sheep were examined by assessing the abundance of OTUs. The results revealed a notable decrease in the Chao1 diversity index in the abomasum of the goats (*p* = 0.04) (Figure 3a). Additionally, the PCoA and NMDS analyses indicated a clear separation of the abomasal microbiota, as well as the colonic microbiota, between the two breeds (Figure 3b,c).

**Figure 4:**
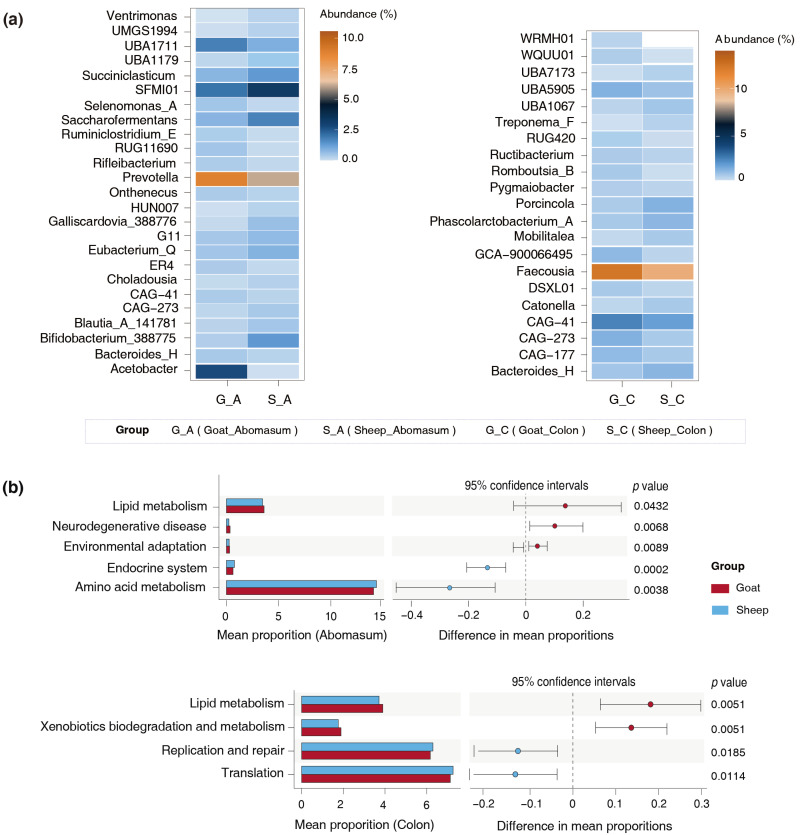
Functional differences in specific bacteria between the goats and sheep. (**a**) Specific bacteria identified from the abomasum and colon of the goats and sheep. (**b**) Functional variations in specific bacteria in the abomasum and colon of the goats and sheep.

Twenty-five specific bacteria in the abomasum and twenty-one in the colon were identified. For example, in the abomasum, the goats had an increase in the abundance of *Prevotella* (*p* = 0.04), *Acetobacter* (*p* = 0.03), and *UBA1711* (*p* = 0.02), but a notable decrease in the abundance of *Bifidobacterium_388775* (*p* = 0.03), *Eubacterium_Q* (*p* = 0.01), *Succiniclasticum* (*p* = 0.02), and *Saccharofermentans* (*p* = 0.01) (Figure 4a). In the colon, the goats presented a lower abundance of *Phascolarctobacterium_A* (*p* = 0.01), *Catonella* (*p* = 0.01), *Porcincola* (*p* < 0.001), and *Treponema_F (p* = 0.04) (Figure 4a). Remarkably, the abundance of *CAG−41* in the abomasum and colon of the goats was significantly higher than that of the sheep. In contrast, *CAG−273* was found at a lower abundance in the abomasum of the goats, but was more prevalent in their colon, suggesting variable distribution across the test sites.

To assess the functional profiles of the specific microbiota, we conducted a phylogenetic analysis of communities by reconstructing unobserved states. The results indicated that the specific bacteria present in the abomasum and colon of Longdong cashmere goats exhibited a greater ability to metabolize lipids (Figure 4b).

### 3.4. Variations in the Concentrations of SCFAs and Lipid-Metabolizing Enzymes in the Gut Chyme of the Goats and Sheep

**Figure 5:**
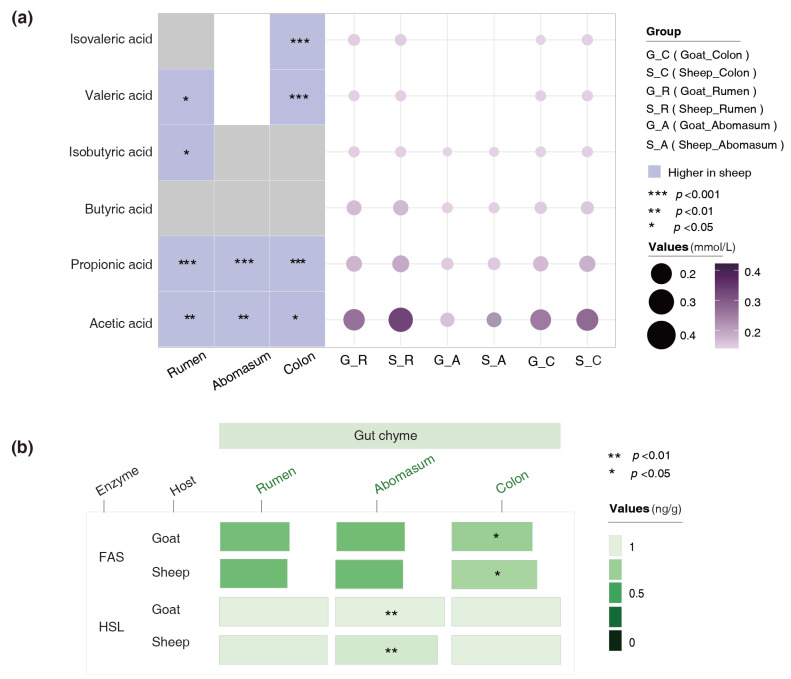
Variations in the concentrations of SCFAs and lipid-metabolizing enzymes in the gut chyme of the goats and sheep. (**a**) Differences in the SCFA content of the gut chyme between the goats and sheep. (**b**) Differences in the FAS and HSL contents of the gut chyme between the goats and sheep.

We determined the concentrations of SCFAs, FAS, and HSL in the ruminal, abomasal, and colonic chyme. The results revealed that the concentrations of acetic acid and propionic acid in all those gut chyme of the goats were significantly lower than those of the sheep. The contents of valeric acid in the abomasal and colonic chyme of the goats were also notably lower than those of the sheep (Figure 5a). The HSL content in the abomasal chyme of the goats was higher than that of the sheep (*p* = 0.004), whereas the FAS content in the colonic chyme was lower than that of the sheep (*p* = 0.01) (Figure 5b). These results suggest that both the host and the microbiota might utilize enzymatic hydrolysis to jointly influence fat deposition in the body.

### 3.5. Association of IMF Deposition with Identified Bacteria and SCFAs

**Figure 6:**
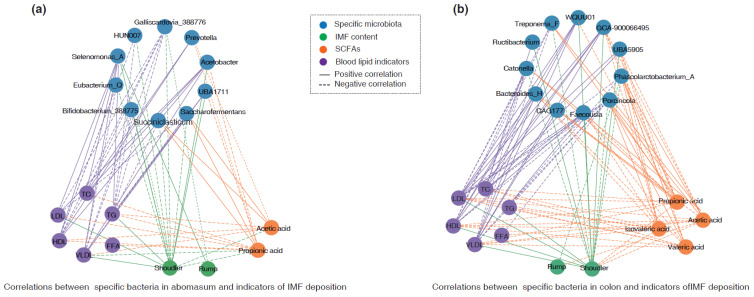
Association of IMF deposition with identified bacteria and SCFAs. (**a**) Correlation network of IMF deposition indicators with specific bacteria and SCFA content in the abomasum. (**b**) Correlation network of IMF deposition indicators with specific bacteria and SCFA content in the colon. In these images, a solid line represents a significant positive correlation, while the dashed line represents a significant negative correlation. Only the results with significantly moderate or strong correlations (|*r*| > 0.45, *p* < 0.05) are displayed.

In the abomasum, a Spearman correlation analysis suggested that the abundance of *Selenomonas_A* was positively correlated with the IMF content in shoulder and rump meats, as well as levels of LDL, TC, and TG (*r* > 0.45, *p* < 0.05) (Figure 6a). The abundance of *Acetobacter* and *UBA1711* was positively correlated with the IMF content in shoulder meat, while it was negatively associated with the concentration of propionic acid (*r* < −0.45, *p* < 0.05). In contrast, the abundance of *Bifidobacterium_388775* (*r* = −0.637, *p* < 0.01), *Galliscardovia_388776* (*r =* −0.721, *p* < 0.001), and *Eubacterium_Q* (*r* = −0.586, *p* < 0.05) was negatively correlated with the IMF content in shoulder meat. The abundance of *Saccharofermentans* and *Succiniclasticum* was negatively correlated with the IMF content in shoulder meat (*r* < −0.45, *p* < 0.05), but positively correlated with the concentrations of acetic acid and propionic acid (*r* > 0.45, *p* < 0.05) (Figure 6a).

Additionally, in the colon, the abundance of *Faecousia, WQUU01, UBA5905,* and *GCA-900066495* was positively associated with the IMF content in shoulder meat and levels of LDL as well as TC (*r* > 0.45, *p* < 0.05), while it was negatively correlated with the concentration of propionic acid (*r* < −0.45, *p* < 0.05). In contrast, the abundance of *Phascolarctobacterium_A, Treponema_F*, *Catonella,* and *Porcincola* was negatively correlated with the IMF content in shoulder meat and level of LDL, while it was positively associated with the concentrations of acetic acid and propionic acid (Figure 6b).

## 4. Discussion

The dressing percentage, GR value, and intramuscular fat content are linked to growth and fat deposition in animals. Variations are perhaps unsurprisingly observed between Longdong cashmere goats and Tan sheep, despite them being co-grazed. Their dressing percentage typically ranged from approximately 40% to 60%, but the sheep exhibited a higher dressing percentage than the goats, which is consistent with Sen et al.’s results [25]. Moreover, contrary to several research findings [25,26] which indicated that goat meat differs from sheep meat in terms of flavor and aroma and tends to be less fatty than mutton, our study revealed that Longdong cashmere goats exhibited a greater capacity for intramuscular fat deposition compared to Tan sheep. Certainly, Casey also reported that the intramuscular fat content of Boer goats was higher than that of four South African sheep breeds [27], which is mostly consistent with our results. These findings indicate that the process of IMF deposition is complex and influenced by various factors, including the age, breed, and diet of the animals [28].

The levels of TG, TC, HDL, LDL, and VDL in serum are closely related to lipid metabolism rates in animals. Studies have shown that the serum levels of TC and TG are positively correlated with IMF content in the muscles [29,30]. This study revealed that the serum levels of TG, TC, FFAs, HDL-C, LDL-C, and VDL-C in the Longdong cashmere goats were notably higher than those in the Tan sheep, and aligned with the observed IMF deposition. This is also essentially in agreement with the findings of Hu et al. [31]. The deposition of IMF is also affected by a series of enzymes. FAS plays a key role in the de novo production of fatty acids, as it can catalyze the formation of fatty acids from malonyl-CoA, hence promoting fat synthesis [32,33]. In contrast, HSL can break down fat into FFA and glycerol, with the FFAs being carried through the bloodstream to provide energy to the body [34,35]. Our findings suggest that the content of FAS in the duodenum of the Longdong cashmere goats was lower, while the content of HSL in the pancreas and duodenum was notably higher. This shift may result in a greater flow of FFAs to adipose tissue, leading to increased IMF deposition in muscle.

We also analyzed microbial community diversity and found that the bacterial diversity of the abomasum and colon of the goats differed from that of Tan sheep, with a reduction in the Chao1 index for the abomasum. Additionally, the analysis of distinct bacteria and functional pathways further validated the idea that the unique bacteria in the goats played a significant role in their lipid metabolism.

It is however important to mention that the enzyme content we assessed in the gut chyme might include enzymes generated by the host. Moreover, there are variations in the types of SCFAs found in the gut chyme of the Longdong cashmere goats compared to Tan sheep, with the amount of acetic acid and propionic acid in the abomasum and colon of the goats being lower than that in the sheep. Acetic acid and propionic acid can enhance energy expenditure and decrease fat deposition by activating the AMP-activated protein kinase [36,37]. Propionic acid can trigger the secretion of the appetite-suppressing hormone glucagon-like peptide-1 from the gastrointestinal tract, leading to a reduction in food consumption and, consequently, less fat accumulation [38,39]. These reports are consistent with our observations that the goats possess a lower SCFA content in their gut chyme.

Our correlation analysis indicated that some specific bacteria may exhibit a moderate correlation with intramuscular fat deposition. For example, *Bifidobacterium_387,352* and *Eubacterium_Q* in the abomasum were negatively related to the intramuscular fat content in shoulder meat. Zhang et al. suggested that a lower abundance of *Bifidobacterium* is associated with increased fat accumulation in sheep [40]. Similarly, other studies have shown that the administration of *Bifidobacterium* can lower levels of TG and TC, as well as reduce fat deposition in mice [41,42,43]. Additionally, *Bifidobacterium* has been shown to degrade polysaccharides, enhance the production and release of insulin and glucagon-like peptide 1, inhibit the absorption of dietary lipids and cholesterol in the small intestine, and further reduce fat deposition [42,44,45]. *Eubacterium-Q* is capable of converting cholesterol into coprosterol, which helps to minimize fat buildup in the host and lower TC levels in the serum and intestines [46,47]. In addition, we identified that *Succiniclasticum*, *Saccharofermentans*, *Treponema_F*, and *Faecousia* were associated with the production of acetic acid, propionic acid, and isovaleric acid, as well as IMF deposition. Previous research has revealed that *Succiniclasticum* can transform succinic acid into propionic acid and generate acetic acid [48,49,50]. *Saccharofermentans*, a common starch-degrading bacterium, can degrade plant polysaccharides to generate acetic acid and propionic acid [51,52]. *Treponema* is capable of producing a majority of SCFAs, particularly acetic acid, propionic acid, and butyric acid [53,54]. Jiao et al. reported that the administration of acetic acid and propionic acid to pigs could reduce lipogenesis and enhance lipolysis in various tissues by regulating hormones and genes, thereby preventing fat deposition in these animals [55].

Our study examines the variation in the IMF deposition of Longdong cashmere goats and Tan sheep, as well as its relationship with gut microbiota. We offer several potential microbes and metabolites that warrant further investigation. Nevertheless, we have not yet fully clarified how the bacteria affect IMF deposition. It will therefore be essential to validate the relationships of the identified bacteria with fat accumulation characteristics in the future. This could include employing germ-free animal models that are colonized with cultured gut bacteria in controlled experimental settings.

## 5. Conclusions

This research revealed variations in dressing percentage, intramuscular fat content in shoulder and rump meat, blood lipid levels, and concentrations of fatty acid synthase and hormone-sensitive lipase in the duodenum of Longdong cashmere goats and Tan sheep, along with their correlations with gut microbiota. Several potential bacteria, including *Acetobacter* and *UBA1711* in the abomasum, as well as *Faecousia*, *WQUU01*, *UBA5905*, and *GCA-900066495* in the colon, were identified for further investigation to explore their effects on intramuscular fat deposition.

## Data Availability

The data presented in this study are available in the NCBI Sequence Read Archive (SRA) database, reference number PRJNA1214480. These data were derived from the following resources available in the public domain: [https://www.ncbi.nlm.nih.gov/bioproject/?term=(PRJNA1214480), accessed on 19 May 2025].
